# Association of stem cell factor gene expression with severity and atopic state in patients with bronchial asthma

**DOI:** 10.1186/s12931-017-0504-2

**Published:** 2017-01-18

**Authors:** Safaa I. Tayel, Sally M. El-Hefnway, Eman M. Abd El Gayed, Gehan A. Abdelaal

**Affiliations:** 1 Medical Biochemistry Department, Faculty of Medicine, Menoufia University, Shebin El-Kom, Egypt; 2 Chest Department, Faculty of Medicine, Menoufia University, Shebin El-Kom, Egypt

**Keywords:** Asthma, Atopy, Steroids, Expression, Stem cell factor

## Abstract

**Background:**

Bronchial asthma is a chronic inflammatory and remodeling disorder of the airways, in which many cells, cellular elements, and cytokines play important roles. Stem cell factor (SCF) may contribute to the inflammatory changes occurring in asthma. We aimed to show the expression of SCF gene in patients with asthma as a means of diagnosis and its association with severity and atopic state in these patients.

**Methods:**

This study was carried out on 80 subjects, 50 asthmatic patients and 30 age and gender matched healthy control persons. They were subjected to full history taking, general and local chest examination, spirometric measurements (pre and post broncodilators) using a spirometer, serum IgE, and real time PCR for assessment of SCF mRNA expression.

**Results:**

This study showed significant difference between the studied groups regarding pulmonary function tests (*P* < 0.001). Asthmatic patients had significant higher SCF expression compared to control (*P* < 0.001), also atopic patients vs non atopic (*P* = 0.03) and severe asthmatic patients vs mild ones (*P* < 0.001). SCF expression at cut off point (0.528) is sufficient to discriminate asthmatic patients from control while at cut off point (1.84) for discrimination of atopic patients from non-atopic patients and at cut off point (1.395) for discrimination of severe asthmatic patients from mild ones. A significant negative correlation between SCF expression and inhaled steroid while significant positive correlation with serum IgE was found.

**Conclusion:**

Measuring SCF mRNA expression can be used as an efficient marker for evaluation of atopy and detection of severity of bronchial asthma.

## Background

Bronchial asthma is a chronic inflammatory and remodeling disorder of the airways, in which many cells, cellular elements, and cytokines play important roles. Many cytokines released by T cells, innate, and structural cells contribute to the different pathogenetic features of asthma [1]. Bronchial asthma is defined by the history of respiratory symptoms such as wheeze, shortness of breath, chest tightness and cough that vary over time and in intensity, together with variable expiratory airflow limitation [2]. Previously published evidence suggested that both environmental components and genetic variations may contribute to asthma susceptibility [3]. The major mast cell growth factor, stem cell factor (SCF) is a cytokine that may contribute to the inflammatory changes occurring in diseases associated with an increased mast cell number and activation, like asthma [4]. The gene for SCF in humans is located on chromosome 12q22-q24 and SCF protein is expressed in two forms soluble form (sSCF) and membrane-bound form (mSCF) [5].

The sSCF and mSCF isoforms are formed by alternative splicing and both are biologically active. SCF^248^ includes exon 6, which encodes a proteolytic cleavage site, resulting in the production of soluble SCF. The lack of exon 6 in human SCF^220^ produces the transmembrane form of human SCF. The ratio of SCF^248^ mRNA to SCF^220^ mRNA varies considerably in different tissues, ranging from 10:1 in the brain and 4:1 in the bone marrow to 0.4:1 in the testis [6].

SCF is expressed in vitro by various cells from the airways, including the bronchial epithelial cells, lung fibroblasts, bronchial smooth muscle cells, endothelial cells, peripheral blood eosinophils and mast cells. Once released, SCF acts on all cells that express the Kit receptor [4]. Human mast cells produce more mSCF than sSCF [7] while pulmonary fibroblasts and smooth muscle cells produce more sSCF than mSCF [8].

SCF initiates its effects by binding to the c-kit receptor, which results in receptor dimerization and activation of multiple signaling pathways; including the Erk1/2 and p38 mitogen-activated protein kinase (MAPK) pathways [9].

The Kit receptor is expressed on cells that respond to SCF, including the haematopoietic progenitor cells and lymphoid lines, melanocytes, germinal cells, eosinophils in the peripheral blood, basophils and mast cells [4].

Signaling from c-Kit is crucial for normal hematopoiesis, pigmentation, fertility, gut movement, and some aspects of the nervous system. Deregulated c-Kit kinase activity has been found in a number of pathological conditions, including cancer and allergy [10]. The stimulation of mast cells by SCF can induce their adhesion to extracellular matrix and degranulation, leading to the production and release of histamine, pro-inflammatory cytokines and chemokines. SCF also induces eosinophil adhesion and activation [11].

SCF acts as an important growth factor for human and murine mast cells [12], including in vitro proliferation and differentiation of immature CD34+ mast cells in the bone marrow [13], and CD34+ progenitors in peripheral blood [14].

We aimed in this study to show the expression of SCF gene in patients with asthma and its association with severity and atopic state in these patients.

## Methods

This case control study was carried out on 80 subjects, 50 asthmatic patients that were admitted in Chest Department, Menoufia University Hospital and 30 age and gender matched healthy control persons. All laboratory investigations and genetic analysis were performed in Medical Biochemistry Department, Faculty of Medicine, Menoufia University. All subjects enrolled in the study (patients and control) were nonsmoker. They were divided into two groups:

**Group 1:** included 50 patients with persistent asthma (24 (48%) males and 26 (52%) females). Their mean age was 33.84 ± 1.46 year. Bronchial asthma was diagnosed clinically based on symptoms and signs plus evidence of expiratory airflow limitation (FEV1/FVC less than 0.7) with positive reversibility test and variability. They were classified into atopic (allergic) 23 (46%) and non-atopic 27 (54%) groups based on the family history, presence of other atopic diseases like allergic rhinitis and dermatitis and by measuring the level of serum IgE by ELISA [1]. Bronchodilator (BD) reversibility test was considered positive if there was an increase in FEV1 of >12% and >200 mL from baseline, 10–15 min after 200 mcg salbutamol inhalation. All patients were on inhaled corticosteroids (budesonide) in a dose varying from 500 to 2000 mg/day according to severity, patients only receive systemic corticosteroid during exacerbations. Patients known to have systemic inflammatory diseases were excluded from this study.

Severity of bronchial asthma in patients was assessed based on the spirometric staging according to the Global Initiative for Asthma (GINA) guidelines 2016 [2] and the National Asthma Education and Prevention Program (NAEPP) classification [15] (Mild: post BD FEV1 ˃ 80% of predicted, Moderate: post BD 60–80% of predicted and severe: post BD ˂ 60% of predicted), patients were classified into mild 16(32%), moderate 20(70%) and severe 14(28%) groups.

**Group 2:** included 30 apparently healthy subjects served as a control group (13 (43.3%) males and 17 (56.7%) females). Their mean age was 35.23 ± 1.83 years.

All patients and control groups were subjected to the following: full history taking, General examination, local chest examination, spirometric measurements (pre and post broncodilators) using a spirometer (Spiroanalyzer ST-90 supplied by Fukuda Sangyo, Tokyo, Japan) and Real time PCR for assessment of SCF mRNA expression.

**Fig. 1:**
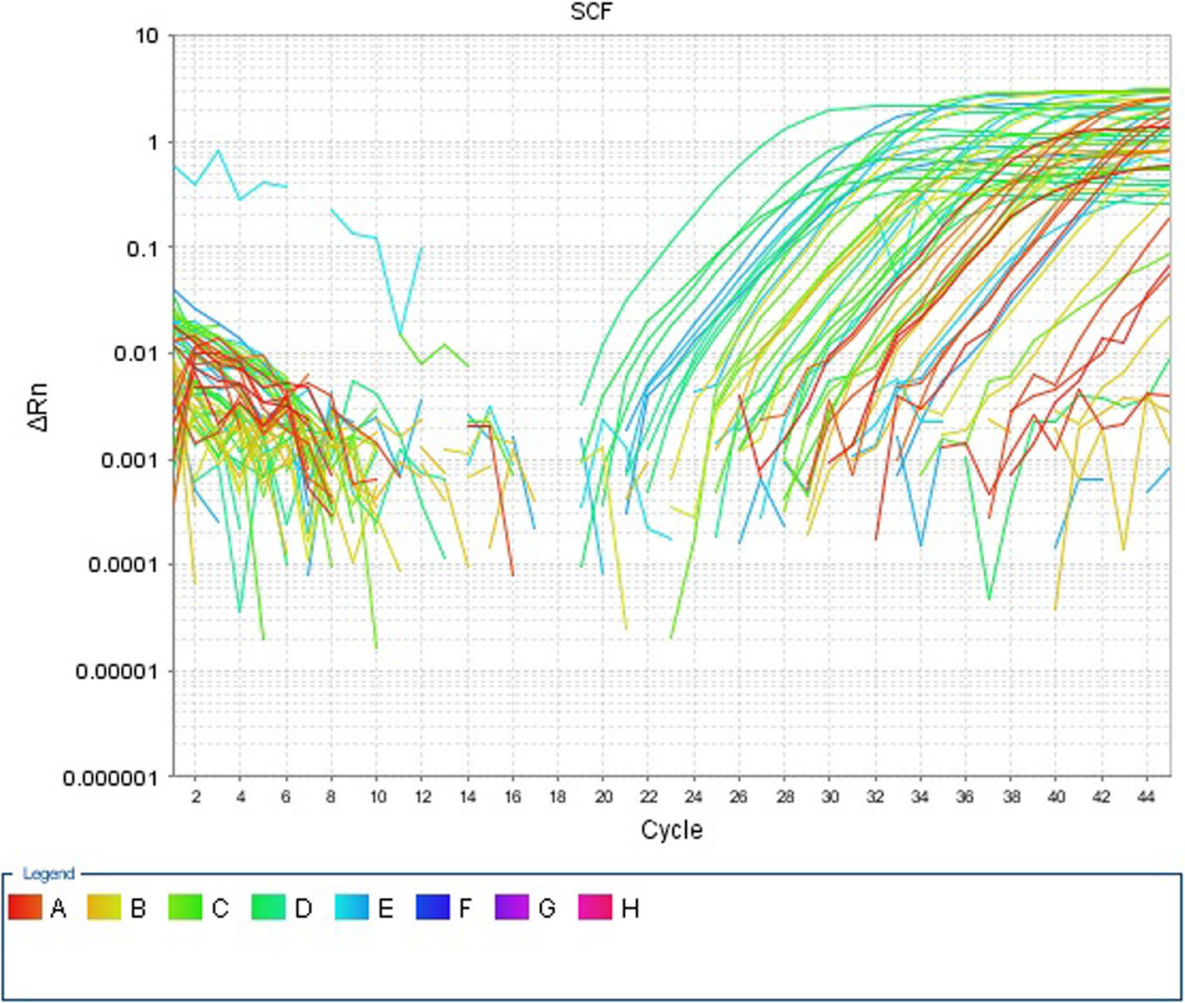
Amplification plot of SCF mRNA gene expression (normalized fluorescence signal (∆Rn) plotted versus cycle number). The plate of real time instrument divided into 96 wells, arranged in 12 columns and 8 rows, the legend represents colors of fluorescence emitted from each 12 samples of 8 rows (from A to H)

**Sample collection and assay:** 2 ml of fresh venous blood was collected from all subjects into EDTA containing tube for estimation of SCF expression by Real time PCR. Quantitative assay of SCF mRNA expression in whole blood using reverse transcriptase polymerase chain reaction (RT-PCR) technique was done as follow: Sample preparation was done at first, then total RNA isolation from whole blood using (Thermo Scientific King Fisher Pure RNA extraction Kit, from Lithuania), followed by assuring RNA quality and purity [16]. Extracted RNA was stored in -80 °C till time of use. First step- PCR was cDNA synthesis (reverse transcription step) using (MyTaq Tm one step RT PCR kit from USA), using Applied Bio systems 2720 thermal cycler (Singapore). Glyceraldehyde 3-phosphate dehydrogenase (GAPDH) primers were used in RT PCR reaction as RNA loading control. Second step- PCR, was cDNA amplification (real time PCR step) (Fig. 1): The cDNA was used in SYBR green based quantitative real-time PCR for quantification of SCF gene expression by (SensiFAST TM SYBR Lo-ROX Kit from USA), using the following designed primers (Midland,Texas). The primers were designed with Oligo Primer Analysis software (Primers detect SCF mRNA that produces both protein isoforms soluble and membrane bound forms of SCF) and the sequences were revised using primer blast (http://www.ncbi.nlm.nih.gov/BLAST/).1-SCF primer sequence:

Forward primer 5′GAGCTCCAGAACAGCTAAACG-3′, Reverse primer 5′- CACTCCACAAGGTCATCCAC -3′ and 2- GAPDH primer sequence: Forward primer 5′ TGATGACATCAAGAAGGTGGTGAAG- 3′, Reverse primer 5′ TCCTTGGAGGCCA TGTGGGC CAT-3′. Data analysis with Applied Biosystems 7500 software version 2.0.1. The relative quantification (RQ) of gene expression performed using comparative ∆∆Ct method [17] where the amount of the target (SCF) mRNA, is normalized to an endogenous reference gene (GAPDH) and relative to a control. Each run was completed using melting curve analysis to confirm specificity of the amplification and absence of primer dimers.

## Results

**Fig. 2:**
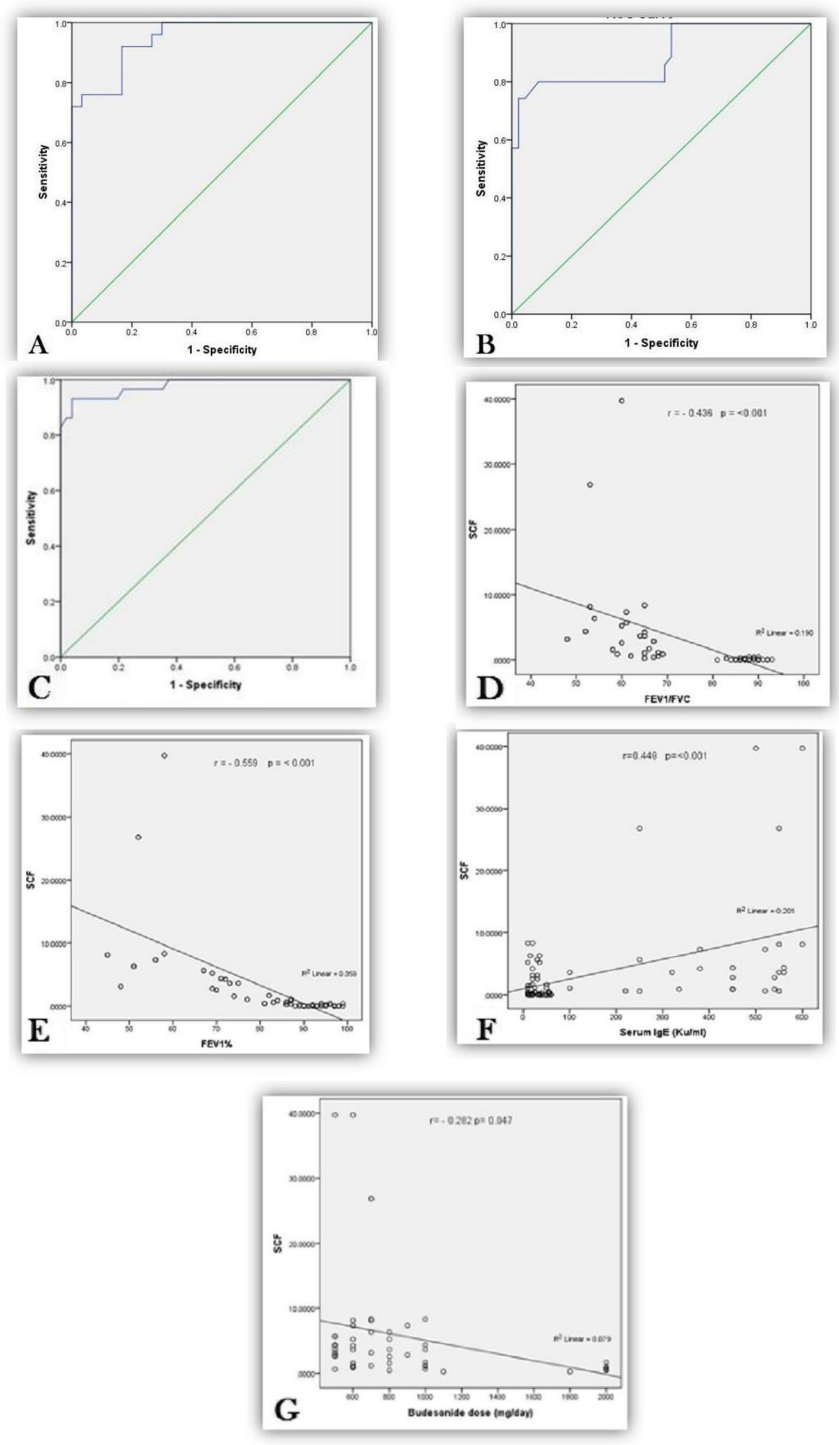
**a** Receiver operating characteristic (ROC) curve of SCF mRNA expression at cut off point (0.528) for discrimination of patients from control, Area under the curve (AUC) = 0.949, Standard error (SE) = 0.022, Confidence interval (CI) = 0.907–0.992. **b** ROC curve of SCF mRNA expression at cut off point (1.84) for discrimination of atopic patients from non-atopic patients, (AUC) = 0.887, SE = 0.039, CI = 0.811–0.964. **c** ROC curve of SCF mRNA expression at cut off point (1.395) for discrimination of severe asthmatic patients from mild asthmatic patients, (AUC) = 0.977, SE = 0.015, CI = 0.948–1.0. **d** Correlation between SCF mRNA expression and FEV1/FVC % in patients group. **e** Correlation between SCF mRNA expression and FEV1% predicted in patients group. **f** Correlation between SCF mRNA expression and serum IgE (Ku/ml). **g** Correlation between SCF mRNA expression and budesonide dose (mg/day)

Results of this study showed that age and gender were matched between studied groups (Table 1). The study also showed high statistical significant difference between studied groups regarding pulmonary function tests (*P* < 0.001) (Table 1). Serum IgE was significantly higher in atopic patients versus non atopic ones (*P* < 0.001) (Table 2). Asthmatic patients had significant higher SCF mRNA expression compared to control (*P* < 0.001), also atopic patients compared with non-atopic patients (*P* = 0.03) and severe asthmatic patients compared with mild asthmatic ones (*P* < 0.001) while SCF mRNA expression was not significant between males and female patients (*P* < 0.05) (Table 3). SCF mRNA expression at cut off point (0.528) is sufficient to discriminate asthmatic patients from control that gives a sensitivity of 92% and a specificity of 84.6% (Table 4, Fig. 2a) while at cut off point (1.84) for discrimination of atopic patients from non-atopic patients with sensitivity of 80% and a specificity of 81.2% (Table 4, Fig. 2b). Furthermore, SCF mRNA expression at cut off point (1.395) can discriminate severe asthmatics from mild asthmatics with sensitivity of 96% and a specificity of 80% (Table 4, Fig. 2c). Moreover, results showed negative correlation between SCF mRNA and pulmonary function tests FEV1/FVC % and FEV1% predicted in asthmatic group (*r* = -0.436 and -0.559) respectively, *p*-value for both (<0.001) (Fig. 2d& e) while significant positive correlation between SCF expression and serum IgE levels (Ku/ml) in asthmatic patients, (*r* = 0.448 and *p*-value <0.001) (Fig. 2f). SCF expression was negatively correlated with inhaled budesonide dose (mg/day) in asthmatic patients, (*r* = - 0.282 and *p*-value <0.047) (Fig. 2g).

**Table 1:** Demographic, clinical data and pulmonary function tests of studied groups

		Patients (*n* = 50)		Control (*n* = 30)		Test	*P*-value
Age in years (Mean ± SEM)		33.84 ± 1.46		35.23 ± 1.83		0.59^a^	0.55
		No	%	No	%		
Gender	Male	24	48%	13	43.3%	0.16^b^	0.68
	Female	26	52%	17	56.7%		
FEV1/FVC %(Mean ± SEM)		61.40 ± 0.80		87.67 ± 0.52		23.52^a^	<0.001
FEV1% predicted (Mean ± SEM)		70.44 ± 1.83		93.47 ± 0.61		9.52^a^	<0.001
Atopy	Atopic	23	46%				
	Non atopic	27	54%				
Severity	Mild	16	32%				
	Moderate	20	70%				
	Severe	14	28%				

Data are presented as mean and standard error mean (SEM)

*FVC* forced vital capacity, *FEV1* Predicted forced expiratory volume in first second

^a^ t -test, ^b^ chi-square (X^2^) test

**Table 2:** Comparison of serum IgE (Ku/ml) between studied groups

	Serum IgE (Ku/ml) (Mean ± SEM)	t- test	*P* value
Patients (*n* = 50)	257.14 ± 32.24	5.37	<0.001
Controls (*n* = 30)	32.37 ± 3.80		
Atopic patients (*n* = 23)	452.88 ± 24.12	14.5	<0.001
Non atopic patients (*n* = 27)	45.08 ± 12.90

**Table 3:** Comparison of SCF mRNA expression between studied groups

	SCF mRNA expression (Mean ± SEM)	Test	*P* value
Patients (*n* = 50)	5.64 ± 1.24	3.35^b^	0.001
Controls (*n* = 30)	0.25 ± 0.027		
Male patients (*n* = 24)	4.05 ± 1.46	0.54^c^	0.60
Female patients (*n* = 26)	3.14 ± 0.9		
Atopic patients (*n* = 23)	8.28 ± 2.44	2.12^b^	0.03
Non atopic patients (*n* = 27)	3.19 ± 0.49		
Mild (*n* = 16)	0.81 ± 0.43	23.19^a^	<0.001
Moderate (*n* = 20)	3.47 ± 1.45		
Severe (*n* = 14)	10.25 ± 2.65		

^a^Kruskal-Wallis test, ^b^ t-test, ^c^ Mann- Whitney U test

**Table 4:** Validity of SCF mRNA expression for discrimination of patients from control, atopic patients from non-atopic patients and severe asthmatic patients from mild asthmatic patients

	Sensetivity	Specificity	Positive predictive value (PPV)	Negative predictive value (NPV)	Accuracy
SCF mRNA expression at cut off point (0.528) for discrimination of patients from control.	92%	84.6%	90.1%	86.2%	88.7%
SCF mRNA expression at cut off point (1.84) for discrimination of atopic patients from non-atopic patients.	80.0%	81.2%	86.9%	70.5%	80%
SCF mRNA expression at cut off point (1.395) for discrimination of severe asthmatic patients from mild asthmatic patients.	96%	80%	88.9%	92.3%	90%

### Statistical analysis

Results were collected, tabulated and statistically analyzed by IBM personal computer and statistical package SPSS version 20. Student t-test used for comparison between two groups having quantitative variables. Chi-square test (χ^2^): was used to study association between two qualitative variables. Mann whitney and Kruskal – Wallis tests for comparison two and three groups of not normally distributed variables respectively. *P*-value < 0.05 was considered statistically significant.

## Discussion

Asthma is a highly prevalent disease involving a complex interplay of environmental factors, airflow obstruction, bronchial hyper-responsiveness, and inflammation. The dominant feature that leads to clinical symptoms is smooth muscle contraction and inflammation, which results in narrowing of the airway and obstruction [18]. Numerous triggers can induce bronchoconstriction, such as allergic responses, respiratory infections, exercise, irritants, and non-steroidal anti-inflammatory drugs [19]. Mast cells and eosinophils are key cells in the inflammatory process ongoing in the airways of patients with asthma [20]. SCF also induces eosinophil adhesion and activation [11].

The present study revealed that pulmonary function tests (FEV1/FVC % & FEV1% predicted) were significantly decreased in asthmatic group when compared to the control group this comes in line with [20] and [21].

In the current study, there was a significant statistical increase of SCF mRNA expression in asthmatic group when compared to controls, Furthermore, this expression is significantly increased in atopic than non-atopic patients. Our observations are in line with Al-Muhsen et al. [22] who reported that, the expression of mRNA for SCF and its receptor c-kit were shown to be higher in the bronchi of patients with asthma as compared to controls. Da Silva and Frossard, [4] also concluded that, SCF expression increases in the airways of asthmatic patients, and this is reversed after treatment with glucocorticoids. SCF may be proposed as an interesting target for asthma treatment through its effect on the regulation of the number and activation status of mast cells. Also, Lei et al., [21] Suggested that allergic asthma is characterized by an elevation of cytokines SCF and IL-31 and the measurement of their expression at either protein level in serum or mRNA level in Peripheral Blood Mononuclear Cells (PBMCs) will be a valuable parameter for the diagnosis of allergic asthma. Makowska et al., [20] found that expression of mRNA for SCF is significantly increased in nasal epithelia of patients with allergic rhinitis.

Kowalski et al., [23] demonstrated increased expression of SCF in cultured epithelial cells. Furthermore, expression of SCF in epithelial cells and the density of mast cells in polyps tissue correlated with the number of polypectomies, suggesting a patho- physiological link between SCF, mast cells and severity of nasal polyposis.

A recent study made by Moaaz and his coworkers [24] demonstrated that the levels of SCF mRNAs in atopic asthmatic patients’ PBMCs were significantly higher than those in controls and nonatopic asthmatics.

Since SCF is the principal growth factor for mast cells, it might be expressed in human bronchi and regulated in airway structural cells in pro- and anti-inflammatory conditions. That is, increased SCF expression in the bronchi of a patient with asthma would be associated with increased number and activation of mast cells [25].

The PBMCs in asthmatic patients express a high level of SCF mRNA, which might be induced by the allergens or asthma-associated factors. The significance of a high level of SCF might be interpreted by the effect of SCF on mast cells such as chemoattracting progenitor mast cells from bone marrow to blood, activating mast cells, thus maintaining the process of asthma [21].

Both mast cells and eosinophils express SCF [26] and its c-Kit receptor at the cell membrane [27]. Salib et al., [28] didnot find the differences in the expression of SCF mRNA between patients with and without allergic rhinitis.

This study revealed a significant direct statistical association between SCF mRNA expression and severity of asthma being higher in patients with severe asthma when compared to patients with mild and moderate asthma with significant negative correlation between SCF mRNA and markers of asthma severity such as FEV1/FVC % and FEV1% predicted in asthmatic group. These findings confirm results obtained by Trudeau et al., [29] who demonstrated that, the expression of mRNA for SCF was shown to be higher in both epithelial and luminal compartments of asthmatics bronchi as compared to controls. There were inverse relationship of epithelial and luminal SCF in relation to asthmas symptoms, which suggest that the loss (sloughing) of SCF expressing cells from the epithelia, confers the greatest risk for severe and poorly controlled asthmas.

El-Gazzar et al., [30] demonstrated that the serum level of SCF was higher in asthmatic patients especially among eosinophilic phenotype than among healthy control subjects. Also there was a significant association between higher SCF and higher levels of asthma severity and blood eosinophil%.

In the present study there was significant positive correlation between SCF expression and serum IgE levels (Ku/ml) in asthmatic patients. This is in accordance with Lei et al., [21] who found that, SCF directly correlate with severity of allergic asthma and it may be useful indicator for allergic asthma providing a new clue for the diagnostic study of allergic asthma, and may be useful for the treatment.

Moaaz and his coworkers [24] revealed a strong correlation between SCF, IL-31 and between both of them and IgE in asthmatics. A direct correlation between SCF, IL-31 and FEV-1/ FVC %, CRP and wheezing existed and suggested that both SCF and IL-31 play an important role in mediating inflammation and enhancing severity of atopic asthma.

Results of this study revealed a significant negative correlation between SCF expression and the inhaled budesonide dose (mg/day) in asthmatic patients. This in agreement with Da Silva et al., [31], Da Silva and Frossard, [4] and Makowska et al., [20] who reported that, the higher expression of SCF in asthmatic bronchi in comparison to healthy controls could be normalized by treatment with inhaled glucocorticosteroids. Glucocorticoids may decrease mast cell number and activation through decreased SCF expression.

Neutralizing SCF with a monoclonal antibody administered by inhalation during antigen provocation inhibits mast cell activation, bronchial inflammation (as shown by the diminution of eosinophil infiltration) [32], and bronchial hyperresponsiveness [33]. Consistent with this finding, intra nasal administration of SCF antisense oligonucleotides decreases lung SCF protein expression in a murine model of asthma, as well as airway hyperresponsiveness, IL-4 production and eosinophil number in the bronchoalveolar lavage [34].

Stem cell factor is a critical factor for hematopoiesis and hematopoietic stem cell survival. It also has a role in mobilization of bone marrow stem cells, as well as in cell differentiation. SCF in conjunction with interleukin-31 may play a significant role in airway remodeling by promoting the recruitment of bone marrow-derived fibroblast precursors in to the lung with the capacity to promote lung myofibroblast differentiation [35].

The results of a recent in vitro assay showed that adult T-cell leukemia stem cells (ATLSCs) cultured with cytokines known to promote stem cell expansion, such as SCF, showed highly proliferative activity and maintained their stem cell fraction. Inhibition of c-kit–SCF signaling with the neutralizing antibody ACK2 affected ATLSC self-renewal and proliferation [36]. Also Kim and his coworkers suggested that inhibition of SCF signaling using cKit inhibitors such as masitinib might provide a novel therapeutic opportunity for the prevention of diabetes-induced retinal vascular leakage [37].

The need for further experiments to elucidate SCF signaling in simple cell culture and use of cKit inhibitors work would be of great value to clarify therapeutic potential of SCF inhibitors in preventing airway inflammation in bronchial asthma.

## Conclusion

Measuring SCF mRNA expression can be used as an efficient marker for differentiation of atopic asthmatic patients from non atopic ones and also can be used for detection of severity of bronchial asthma. Moreover, it can be used for evaluation of response to inhaled budesonide therapy in asthma.

## Abbreviations

BD: Bronchodilator
GAPDH: Glyceraldehyde 3-phosphate dehydrogenase
GINA: Global Initiative for Asthma
MAPK: Mitogen-activated protein kinase
NAEPP: National Asthma Education and Prevention Program
RT-PCR: Reverse transcriptase polymerase chain reaction
SCF: Stem cell factor

## References

[1] Willart M, Hammad H (2011). Lung Dendritic cell-epithelial cell crosstalk in Th2 responses to allergens. Curr Opin Immunol.

[2] Global Initiative for Asthma (GINA), Global strategy for asthma management and prevention, update 2016. Available from: <http://www.ginasthma.org/>.

[3] Temesi G, Virag V, Hadadi E (2014). Novel genes in human asthma based on a mouse model of allergic airway inflammation and human investigations. Allergy Asthma Immunol Res..

[4] Da Silva CA, Reber L, Frossard N (2006). Stem cell factor expression, mast cells and inflammation in asthma. Fundamentals and clinical pharmacology.

[5] Anderson DM, Williams DE, Tushinski R (1991). Alternate splicing of mRNA encoding human mast cell growth factor and localization of the gene to chromosome12q22-q24. Cell Growth Differ..

[6] Huang EJ, Nocka KH, Buck J, Besmer P (1992). Differential expression and processing of two cell associated forms of kit-ligands KL-1 and KL-2. Mol Biol Cell..

[7] Welker P, Grabbe J, Gibbs B, Zuberbier T, Henz BM (1999). Human mast cells produce and differentially express both soluble and membrane-bound stem cell factor. Scand. J. Immunol..

[8] Kiener HP, Hofbauer R, Tohidast-Akrad M (2000). Tumor necrosis factor alpha promotes the expression of stem cell factor in synovial fibroblasts and their capacity to induce mast cell chemotaxis. Arthritis Rheum.

[9] Rönnstrand L (2004). Signal transduction via the stem cell factor receptor/c-Kit. Cell Mol Life Sci..

[10] Lennartsson J, Ronnstrand L (2012). Stem cell factor receptor/c-Kit: from basic science to clinical implications. Physiol. Rev.

[11] Okayama Y, Kawakami T (2006). Development, migration, and survival of mast cells. Immunol Res.

[12] Galli SJ, Tsai M, Wershil BK, Tam SY, Costa JJ (1995). Regulation of mouse and human mast cell development, survival and function by stem cell factor, the ligand for the c-kit receptor. Int. Arch. Allergy Immunol..

[13] Kirshenbaum AS, Goff JP, Kessler SW, Mican JM, Zsebo KM, Metcalfe DD (1992). Effect of IL-3 and stem cell factor on the appearance of human basophils and mast cells from CD34+ pluripotent progenitor cells. J. Immunol..

[14] Rottem M, Okada T, Goff JP, Metcalfe DD (1994). Mast cells cultured from the peripheral blood of normal donors and patients with mastocytosis originate from a CD34+/ Fcepsilon RI-cell population. Blood.

[15] National Heart Lung and Blood Institute. Washington DC: Department of Health and Human Services. National Asthma Education and Prevention Program. Expert Panel Report 3: Guidelines for the Diagnosis and Management of Asthma. 2007

[16] Wang E, Miller L, Ohnmacht G, Liu E, Marincola F (2000). High-fidelity mRNA amplification for gene profiling. Nature Biotechnology..

[17] Dorak M (2000). Real-time PCR. Clinical Chemistry..

[18] Ishmael F (2011). The Inflammatory Response in the Pathogenesis of Asthma. JAOA.

[19] Barnes PJ (2008). Immunology of asthma and chronic obstructive pulmonary disease [review]. Nat Rev Immunol.

[20] Makowska J, Cieslak M, Kowalski M (2009). Stem cell factor and its soluble receptor (c-kit) in serum of asthmatic patients- correlation with disease severity. Pulmonary Medicine.

[21] Lei Z, Liu G, Huang Q, Lv M, Zu R, Zhang GM, Feng ZH, Huang B (2008). SCF and IL-31 rather than IL-17 and BAFF are potentialindicators in patients with allergic asthma. Allergy.

[22] Al-Muhsen SZ, Shablovsky G, Olivenstein R, Mazer B (2004). Q H: The expression of stem cell factor and c-kit receptor in human asthmatic airways. Clin Exp Allergy.

[23] Kowalski ML, Lewandowska-Polak A, Woźniak J, Ptasiñska A, Jankowski A (2005). Wagrowska-DanilewiczM, Danilewicz M, Pawliczak R: Association of stem cell factor expression in nasal polyp epithelial cells with aspirin sensitivity and asthma. Allergy..

[24] Moaaz M, Abo El-Nazar S, Abd El-Rahman M, Soliman E (2016). Stem Cell Factor and Interleukin-31 Expression: Association with IgE among Egyptian Patients with Atopic and Nonatopic Bronchial Asthma. Immunol Invest..

[25] Da Silva CA, Frossard N (2005). Regulation of stem cell factor expression in inflammation and asthma. Mem Inst Oswaldo Cruz, Rio de Janeiro.

[26] Hartman M, Piliponsky AM, Temkin V, Levi-Schaffer F (2001). Human peripheral blood eosinophils express stem cell factor. Blood.

[27] Yuan Q, Austen KF, Friend DS, Heidtman M, Boyce JA (1997). Human peripheral blood eosinophils express a functional c-kit receptor for stem cell factor that stimulates very late antigen4 (VLA-4)-mediated cell adhesion to fibronectin and vascular cell adhesion molecule1 (VCAM-1). J. Exp. Med..

[28] Salib RJ, Kumar S, Wilson SJ, Howarth PH (2004). Nasal mucosal immunoexpression of the mast cell chemoattractants TGF-beta, eotaxin, and stem cell factor and theire receptors in allergic rhinitis. J Allergy Clin Immunol.

[29] Trudeau J, Fajt M, Wenzel S : Loss Of Stem Cell Factor Expressing Epithelial Cells To The Airway Lumen: A Risk For Severe And Poorly ControlledAsthma.2012; ChapterDOI:10.1164/ajrccmconference.

[30] El-Gazzar A, Abd Alsamea S, Mohamad O, Khamis A, El-Naggar M (2016). Study of the level of stem cell factor in patients with bronchial asthma. Egyptian Journal of Chest Diseases and Tuberculosis.

[31] Da Silva CA, Kassel O, Lebouquin R, Lacroix EJ, Frossard N (2004). Paradoxical early glucocorticosteroid induction of stem cell factor (SCF) expression in inflammatory conditions. Br J Pharmacol.

[32] Lukacs NW, Kunkel SL, Strieter RM (1996). The role of stem Cell factor (c kit ligand) and inflammatory cytokines in pulmonary mast cell activation. Blood.

[33] Campbell E, Hogaboam C, Lincoln P, Lukacs NW (1999). Stem cell factor-induced airway hyperreactivity in allergic and normal mice. Am. J. Pathol.

[34] Finotto S, Buerke M, Lingnau K, Schmitt E, Galle PR, Neurath MF (2001). Local administration of antisense phosphoro-thioateoligonucleotides to the c kit ligand, stem cell factor, suppresses airway inflammation and IL-4 production in a murine model of asthma. J. Allergy Clin. Immunol.

[35] Dolgachev VA, Ullenbruch MR, Lukacs NW, Phan SH (2009). Role of Stem Cell Factor and Bone Marrow-Derived Fibroblasts in Airway Remodeling. The American Journal of Pathology.

[36] Kuribayashi W, Takizawa K, Sugata K, Kuramitsu M, Momose H, Sasaki E, Hiradate Y, Furuhata K, Asada Y, Iwama A, Matsuoka M, Mizukami T, Hamaguchi I. Impact of the SCF signaling pathway on leukemia stem cell-mediated ATL initiation and progression in an HBZ transgenic mouse model. Oncotarget. 2016;7(32):51027-43. 10.18632/oncotarget.10210 PMC5239456 27340921

[37] Kim SR, Im JE, Jeong JH, Kim JY, Kim JT, Woo SJ, Sung JH, Park SG, Suh W (2016). The cKit Inhibitor, Masitinib, Prevents Diabetes-Induced Retinal Vascular Leakage. Invest Ophthalmol Vis Sci.

